# Clinical management of women in BRCAX families: issues and controversies

**DOI:** 10.1186/1897-4287-10-S2-A1

**Published:** 2012-04-12

**Authors:** G Mitchell

**Affiliations:** 1 Family Cancer Clinic, Peter MacCallum Cancer Centre, East Melbourne, 3002, Australia

## 

The role of the familial cancer clinic (FCC) is to provide a cancer risk assessment and appropriate cancer risk management advice, but there are certain groups of patients for whom there are no standard risk management guidelines. One such group is women with a strong family history of breast cancer but BRCA genetic testing has not found a germline mutation. As a family history of breast cancer is the commonest reason for referral to FCCs, this clinical scenario is a frequent challenge to us all.

This presentation will provide a summary of the literature surrounding:

• Breast cancer risk in BRCAX families

• Ovarian cancer risk in BRCAX families

• Does the presence of male breast cancer affect the breast and ovarian cancer risks for women in BRCAX families

